# Sunburn Awareness and Post‐Exposure Symptoms in Self‐Identified People of Color: A Cross‐Sectional Survey

**DOI:** 10.1111/ijd.70340

**Published:** 2026-02-13

**Authors:** Briana Williams, Liliana Guerrero, Fabiola Moreno Echevarria, Jennifer Shastry, Roopal V. Kundu

**Affiliations:** ^1^ Northwestern University Feinberg School of Medicine Chicago Illinois USA

**Keywords:** Fitzpatrick skin phototype, general dermatology, people of color, skin of color, skin tone, sunburn, UV exposure

Although people of color (POC) experience a lower incidence of skin cancer, morbidity and mortality are often higher due to delayed diagnosis and underrecognition of sun injury [1, 2]. Sunburn is a common precursor to ultraviolet (UV) damage; however, how sunburn symptoms are perceived and labeled among POC remains poorly studied. This cross‐sectional survey assessed sunburn awareness and post‐sun exposure symptoms among POC. Recognizing the limitations of current terminology, phenotype‐based skin classification systems, and substantial heterogeneity of skin tone within racial/ethnic groups [3], participant inclusion as POC was based on self‐identification rather than exclusion by race or ethnicity.

English‐speaking U.S. adults were recruited via ResearchMatch and large public Facebook community groups to complete an online REDCap questionnaire assessing demographics, self‐identified race/ethnicity, self‐reported skin tone using the Monk Skin Tone (MST) scale, and Fitzpatrick skin phototype. Participants were asked, “Have you ever experienced a sunburn?” with options frequently, sometimes, rarely, or never. Participants were also asked, “Have you experienced any of the following after sun exposure?” with options including redness, peeling, scaliness, dryness, blistering, pain on touch, itching, irritation, headaches, hot or warm skin, development of darker skin, or none. Additional items assessed tanning potential and dermatology visit history.

Among 2330 ResearchMatch respondents, 64% (1498) accepted the invitation. 1069 (71%) participants completed the survey. Demographics were comparable to ResearchMatch and U.S. census benchmarks across age, race, and sex. Data were analyzed in R using chi‐square and Fisher's exact tests.

Among 1069 respondents, 372 (35%) self‐identified as POC: 184 Black or African American, 76 mixed, 61 Asian, 31 White, 6 Native American, 2 Pacific Islander, and 12 other. Hispanic/Latino ethnicity was reported by 69 POC respondents, including individuals identifying as mixed race (*n* = 44) and White (*n* = 25). All MST tones (1–10) were represented among POC (mean 5.29, SD 1.66), though 33% (123) felt the MST scale did not accurately capture their tone.

POC who frequently experienced sunburns had lower average MST values (4.63 ± 2.08) than those who never experienced a sunburn (6.45 ± 1.35). Notably, 15% of POC (56/372) reported never having had a sunburn but endorsed post‐sun exposure symptoms, compared with 0.5% of non‐POC (3/663; *p* < 0.001) (Table 1). Common symptoms included hot/warm skin, headache, dryness, itchiness, and peeling.

**TABLE 1:** Discrepancy between self‐reported sunburn history and post‐sun exposure symptoms in POC vs. non‐POC.

Outcome/symptom	POC % (*n*)	Non‐POC % (*n*)	*p*
Never had sunburn but reported symptoms	15% (56/372)	0.5% (3/663)	< 0.001
Hot/warm skin	54% (30/56)	33% (1/3)	—
Headaches	29% (16/56)	—	—
Dryness	27% (15/56)	—	—
Itchiness	14% (8/56)	33% (1/3)	—
Peeling	11% (6/56)	—	—
Redness/inflammation	7% (4/56)	33% (1/3)	—

Abbreviation: POC, people of color.

Analysis by MST categories revealed that lighter tones reported more pain to touch and peeling, medium tones more skin darkening and warmth, and lighter and medium tones more redness/inflammation compared to deeper tones (Table 2). Additionally, POC were less likely than non‐POC to have annual skin exams (11% vs. 38%; *p* < 0.001) and more likely to have never been examined by a dermatologist (40% vs. 21%; *p* < 0.001).

**TABLE 2:** Post‐sun exposure symptoms by Monk Skin Tone category in people of color.

Symptom	Light tones (1–4) % (*n*)	Medium tones (5–6) % (*n*)	Deep tones (7–10) % (*n*)	*p*
Pain to touch	46% (57)*	22% (33)	18% (17)*	< 0.001
Peeling	64% (79)*	47% (71)	28% (27)*	< 0.001
Development of darker skin	55% (68)	64% (96)*	69% (67)*	0.038
Hot/warm skin	56% (70)	50% (76)*	49% (48)*	0.020
Redness/inflammation	69% (86)*	47% (71)*	19% (18)**	< 0.001

* Statistically significant difference compared with one other group (*p* < 0.05).

** Statistically significant difference compared with light and medium tones (*p* < 0.05).

These findings highlight a discordance between how individuals, particularly those with darker skin tones, label sunburn and how they experience symptoms following sun exposure. Some symptoms (e.g., hot/warm skin, headache) may reflect systemic heat exposure or dehydration rather than cutaneous photoinjury. However, symptoms more likely associated with cutaneous UV damage, including erythema, pruritus, tenderness, peeling, and dryness, may not have been labeled as sunburn, reflecting differences in symptom recognition and terminology rather than the absence of cutaneous photoinjury.

These findings align with prior work demonstrating that Fitzpatrick phototype and traditional sunburn descriptors perform poorly in individuals with skin of color [4]. Recent expert consensus highlights persistent gaps in photoprotection education for skin of color populations among both patients and clinicians [5]. Variation in symptom profiles across skin tones underscores the need for education materials that accurately depict sun damage in diverse populations. Limitations include self‐reported data, recall bias, and small subgroup sizes. Improving awareness of sunburn presentation across diverse tones may enhance early recognition and reduce UV‐related morbidity.

## Funding

The authors have nothing to report.

## Ethics Statement

Reviewed and determined IRB exempt by Northwestern IRB; ID #00221410.

## Consent

Consent for publication of the de‐identified survey data was obtained by the authors, and all participants gave consent with the understanding that this information may be publicly available.

## Conflicts of Interest

The authors declare no conflicts of interest.

## Data Availability

The data that support the findings of this study are available from the corresponding author upon reasonable request.
